# Skull Pneumatization Forms a Biothermal System Protecting Ocular and Vestibular Homeostasis

**DOI:** 10.3390/jcm15114259

**Published:** 2026-05-31

**Authors:** Elad Avraham, Israel Melamed

**Affiliations:** Department of Neurosurgery, Soroka University Medical Center, Ben-Gurion University of the Negev, Beer-Sheva 8457108, Israel; melamedi@bgu.ac.il

**Keywords:** paranasal sinuses, mastoid air cells, skull pneumatization, thermoregulation, vitreous, endolymph, vestibular, ocular

## Abstract

**Background:** Paranasal sinuses and mastoid air cells have been attributed to multiple functions—such as voice resonance, cranial lightening, and pressure regulation—yet their potential role in local thermal homeostasis remains underappreciated. The thermoregulatory hypothesis, first proposed in the mid-twentieth century, was largely abandoned after the mid-century, when anthropological findings of climate-correlated variation seemed contradictory. **Hypothesis:** We propose that pneumatized skull regions form a three-component craniofacial biothermal system that maintains thermal stability in the ocular vitreous and vestibular endolymph, two avascular, temperature-sensitive structures that lack intrinsic thermoregulatory capacity. This represents a novel integration that explicitly links paranasal and mastoid pneumatization into a coordinated system that protects sensory organs, distinct from previous brain-cooling hypotheses. **Mechanism:** The system comprises: (1) passive thermal insulation via air spaces, providing ~15-fold greater thermal resistance than bone; (2) active cold protection via mucosal heat delivery (estimated 2–5 W capacity); and (3) active heat dissipation via evaporative cooling (estimated 0.3–0.5 W capacity). This architecture provides asymmetric protection, with cold buffering exceeding heat dissipation by approximately 5- to 15-fold, consistent with thermodynamic constraints and putative evolutionary priorities. **Evidence:** Preliminary observations consistent with this hypothesis include the anatomical proximity of pneumatized regions to the vitreous and labyrinth, intranasal selective brain cooling studies, and clinical observations after mastoidectomy showing preserved pressure buffering but reduced vestibular thermal insulation under extreme stimulation. Climate-correlated pneumatization patterns are consistent with bidirectional thermal adaptation. **Implications:** We present five falsifiable predictions that can be tested with thermographic imaging, pharmacological manipulation, and computational modeling. Validation could inform surgical planning, explain postoperative thermal-sensitivity symptoms, and provide evolutionary insights into craniofacial adaptation.

## 1. Introduction

### 1.1. The Enduring Puzzle of Skull Pneumatization

The human skull contains several interconnected air-filled spaces: the paired paranasal sinuses (frontal, ethmoid, sphenoid, and maxillary) and the mastoid air cell system within the temporal bone. Traditional explanations include voice resonance, skull weight reduction, pressure regulation, and airflow conditioning. However, none fully account for three key observations: (1) significant individual variation: healthy adults show 10-fold differences in total sinus volume; (2) climate-correlated population differences: Arctic populations display smaller frontal sinuses, while equatorial populations show greater pneumatization; and (3) evolutionary persistence: why maintain infection-prone air cavities across diverse environments?

### 1.2. Historical Context and Reappraisal

The thermal function hypothesis has deep historical roots. Proetz (1953) proposed that sinuses serve as “thermal jackets” that protect vital cranial structures [1]. However, anthropological studies by Koertvelyessy (1972) [2] and Negus (1958) [3] found that Arctic populations showed reduced frontal sinus pneumatization compared with warm-climate populations, seemingly contradicting the thermal function hypothesis.

This interpretation may have been premature. A bidirectional thermoregulatory model resolves the apparent contradiction: large sinuses facilitate heat dissipation in warm climates (acting as evaporative radiators), whereas small sinuses minimize heat loss in cold environments (reducing thermal windows). Climate-adaptive patterns are consistent with rather than directly supporting thermal function; phylogenetic confounding and population history remain alternative explanations (see Section 4.7).

Recent work has revived interest in specific aspects of thermal function. Magnuson (2003) proposed that the mastoid autoregulates middle ear temperature [4]. Gallup and Hack (2011) hypothesized that the sinuses act as “brain radiators” [5]. Švagan et al. (2025) reported clinical observations after mastoidectomy consistent with a thermal buffering role: mastoid function testing demonstrated preserved pressure-buffering capacity but reduced thermal insulation of the vestibular organ under extreme thermal stimulation, with the authors characterizing the long-term clinical impact as minor [6].

### 1.3. The Novel Integration: What Has Been Missing

A unified framework that integrates the sinuses and mastoid into a coordinated system protecting sensory organs has been lacking. Previous work has focused on brain cooling [5], mastoid function in isolation [4], or general anatomical observations [1]. None have explicitly addressed the unique vulnerability of avascular sensory structures—vitreous and endolymph—which lack intrinsic thermoregulatory capacity yet require precise thermal stability.

This hypothesis proposes the following: (1) The avascular properties of the vitreous and endolymph may make them important sites for pneumatic thermal protection, potentially more so than the brain; (2) pneumatization involves three mechanistically distinct components, rather than serving solely as an insulating process; and (3) an integrated model considering both the sinuses and mastoid region generates quantitative and testable predictions.

## 2. Anatomical Background

### 2.1. Paranasal Sinuses: The Periorbital Thermal Envelope

**Figure 1:**
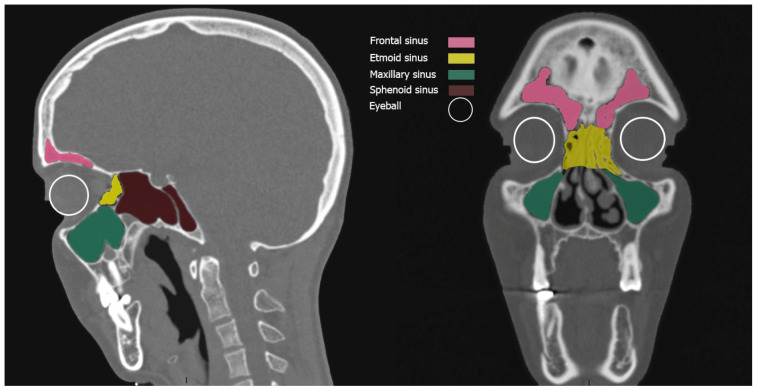
Anatomical relationship between paranasal sinuses and the orbital cavity. CT images in sagittal (**left**) and coronal (**right**) planes show the distribution of paranasal sinuses around the orbit. Color overlays indicate the frontal sinus (pink), ethmoid air cells (yellow), maxillary sinus (teal), and sphenoid sinus (brown). White circles mark the position of the eyeball, illustrating the nearly complete air-space enclosure surrounding the orbit (270–300 degrees of circumferential coverage). Note the close proximity of the ethmoid air cells to the medial orbital wall (lamina papyracea, 0.2–0.5 mm thick). The CT images shown are de-identified institutional scans used retrospectively with institutional ethics approval. The orbital diameter (approximately 35–40 mm) provides anatomical scale reference. The green letter R denotes the patient’s right side (standard radiological orientation marker).

The paranasal sinuses create a nearly complete air-space enclosure around the orbital cavity. The frontal sinus is located above the orbit (3–10 cm^3^); the ethmoid air cells are medial to the orbit, separated by the paper-thin lamina papyracea (0.2–0.5 mm); the maxillary sinus is below (10–20 cm^3^); and the sphenoid sinus is behind, close to the optic nerve (2–5 mm apart). The total mucosal surface area ranges from 150 to 200 cm^2^, with an overall air volume of 30–40 cm^3^. This configuration offers about 270–300° of circumferential coverage of the orbital cavity [7] (Figure 1).

### 2.2. Mastoid Air Cells: The Labyrinthine Thermal Shield

**Figure 2:**
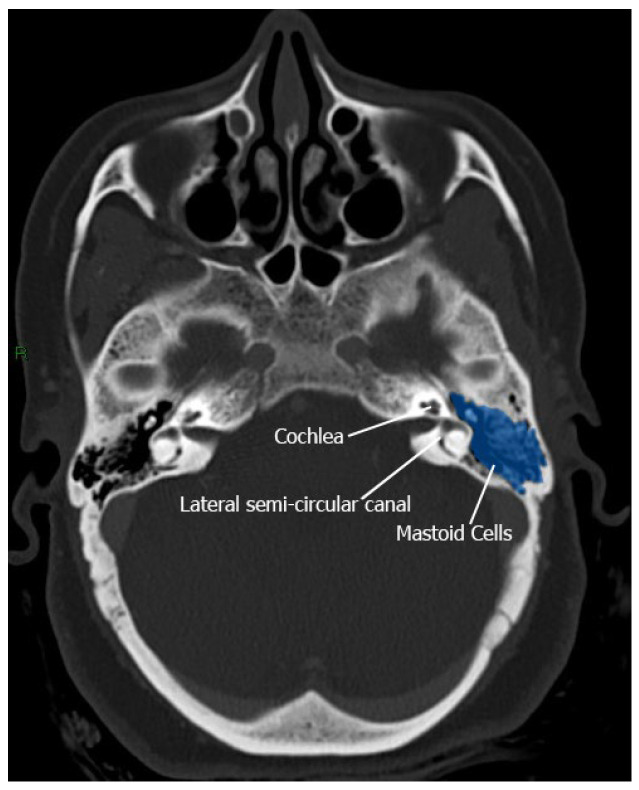
Mastoid air cells and their relation to vestibular structures. An axial CT image at the level of the temporal bone shows the mastoid air cell system (highlighted in blue on the left side). The labeled key anatomical landmarks include the cochlea and lateral semicircular canal, demonstrating the close proximity (2–8 mm) between the pneumatized mastoid and the vestibular labyrinth, which contains temperature-sensitive endolymph. The CT image shown is a de-identified institutional scan used retrospectively with institutional ethics approval. The cochlea (approximately 7–9 mm in diameter) provides anatomical scale reference.

The mastoid is made up of a honeycomb network of air cells within the temporal bone (total volume 2–20 cm^3^, which varies greatly). The system connects to the middle ear through the aditus ad antrum, lined with the respiratory epithelium. Its mucosal surface area is about 100 cm^2^, with a 2–8 mm gap to the vestibular labyrinth through the otic capsule. The sigmoid sinus, a major vein, is located immediately next to it [8] (Figure 2).

### 2.3. Target Structures: Vitreous and Endolymph

The ocular vitreous (4 mL, avascular gel) and vestibular endolymph (0.2 mL, avascular fluid) share a key trait: their lack of vascular perfusion means they cannot regulate their own temperature. The vitreous fills the optical path and needs stable refractive properties; temperature changes can affect its viscosity and refractive index. Endolymph sensitivity to temperature changes is shown clinically: caloric testing uses only 7 °C temperature differences to provoke strong vestibular responses within 20–40 s [9].

## 3. Proposed Mechanism: Three-Component Architecture

*Critical distinction:* This hypothesis presents three functionally distinct components, each with different physical mechanisms, regulatory control, and capacity limits. This tripartite architecture distinguishes our framework from previous models and may explain observations that simpler models cannot accommodate. Importantly, passive thermal insulation is an inherent physical consequence of air-filled spaces, not necessarily a selected adaptation; the novel adaptive claims of this hypothesis concern Components 2 and 3 and their coordination. Full derivations and sensitivity analyses for all calculations are available in Supplementary Materials S1.

### 3.1. Component 1: Passive Thermal Insulation

**Physical mechanism:** Air-filled spaces provide low-conductivity barriers that impede heat flow equally in both directions. Air’s thermal conductivity (k = 0.026 W/m·K at 37 °C) is approximately 15-fold lower than that of cortical bone (k = 0.32–0.47 W/m·K) [10,11]. For equivalent thickness, this yields ~15-fold greater thermal resistance (a range of 12- to 18-fold across physiological bone conductivity values; see Supplementary Materials S1, Section S3). Heat flux reduction approaches 93% compared with solid bone of equal thickness.

**Key characteristics:** It is always active (structural), symmetric (blocks both directions), predictable (obeys Fourier’s law), and physiologically independent. This component provides baseline buffering that persists even when active mechanisms are compromised.

### 3.2. Component 2: Active Cold Protection

**Physiological mechanism:** When ambient temperature drops, mucosal blood flow may increase, delivering core body heat to the sinus and mastoid air spaces. The respiratory system warms inspired air from ambient temperature (potentially −20 °C) to nasopharyngeal temperature (32–34 °C), resulting in a total heat transfer of 7–11 W during cold exposure [12]. The sinonasal contribution is estimated at 20–40% of this total (see Supplementary Materials S1, Section S4.2 for justification), yielding approximately 2–5 W capacity (estimated range 1.4–4.4 W). This 20–40% fraction is the main source of quantitative uncertainty in Component 2 estimates; the calculation is an approximation rather than a directly measured value.

**Key characteristics:** It requires metabolic energy, is autonomically regulated, can modulate 3–5× via vasoactive control, and is mainly limited by cardiac output. This describes active heating, similar to a radiator delivering heat to a space, not just insulation.

### 3.3. Component 3: Active Heat Dissipation

**Physiological mechanism:** During heat stress, mucosal evaporation and venous pre-cooling can remove excess heat. Evaporation from mucosal surfaces absorbs latent heat (2.4 kJ/g). Estimated sinonasal evaporation of 10–20 mL per day produces approximately 0.3–0.5 W of continuous heat removal. Additional cooling may happen through venous blood returning via the cavernous sinus, although the efficiency of countercurrent exchange in humans remains uncertain [13].

**Key characteristics:** It needs water (impaired when dehydrated), requires a humidity gradient (ineffective above 90% RH), and has a limited surface area. These factors primarily limit heat dissipation capacity.

### 3.4. Asymmetric Architecture: Thermodynamic Rationale

**Figure 3:**
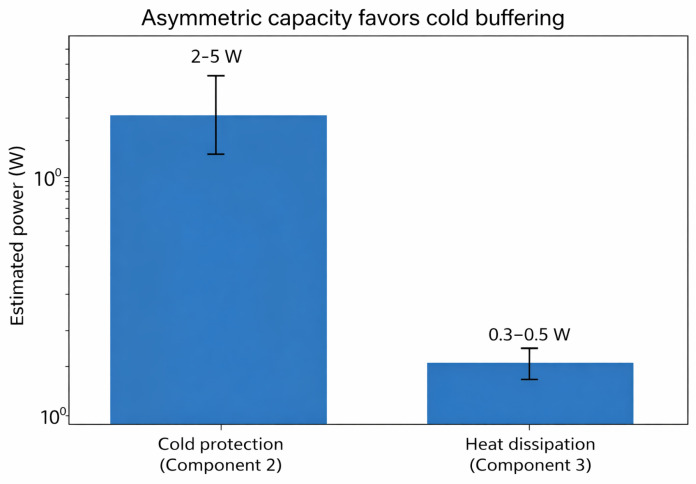
Asymmetric capacity favors cold buffering. Bar graph comparing the estimated thermal protection capacities of the two active components. Cold protection (Component 2, left bar) provides 2–5 W of capacity through mucosal heat delivery, while heat dissipation (Component 3, right bar) offers only 0.3–0.5 W through evaporative cooling. This 5- to 15-fold difference reflects key thermodynamic constraints: namely, larger temperature gradients during cold stress and abundant core metabolic heat compared to surface-limited evaporative capacity. Error bars show estimated parameter uncertainty ranges. Note: logarithmic scale on Y-axis.

Cold-protection capacity (2–5 W) exceeds heat dissipation (0.3–0.5 W) by approximately 5- to 15-fold (conservative central estimate; the full calculated range across parameter uncertainty is 4- to 17-fold, with sensitivity analysis in Supplementary Materials S1, Section S6; Section S10 contextualizes these capacities against estimated orbital thermal loads of 0.12–1.3 W under realistic conditions). This asymmetry results from fundamental thermodynamics: temperature gradients during cold stress (40–60 °C from core to ambient) greatly surpass those during heat stress (5–10 °C). Additionally, core metabolic heat is abundant, while evaporative capacity is surface-limited. Sensitivity analysis shows this asymmetry remains consistent across physiologically plausible parameter ranges (Figure 3).

**Evolutionary interpretation (speculative):** Preventing hypothermia in temperature-sensitive neural and sensory tissues might have been more vital than controlling mild hyperthermia because behavioral thermoregulation (seeking shade, reducing activity) works better against heat but is less effective for cold. Natural selection may have favored systems with a high capacity for cold buffering.

## 4. Discussion

### 4.1. Clinical Evidence: Post-Mastoidectomy Thermal Sensitivity

Švagan et al. (2025) [6] conducted a retrospective cohort study of 30 adults with unilateral childhood mastoidectomy performed for acute coalescent mastoiditis, comparing the operated ear with the intact contralateral ear as a paired within-subject control. Mastoid function testing demonstrated preserved pressure-buffering capacity but reduced thermal insulation of the vestibular organ under extreme thermal stimulation after mastoidectomy. Reported between-ear differences included ΔVOR gain over 0–12 min (*p* = 0.033) and differences in mastoid temperature parameters (T_M and ΔT_M, *p* = 0.022–0.045). The authors characterized the long-term clinical impact as minor or negligible. Despite this, the dissociation of preserved pressure regulation from reduced thermal insulation under non-physiological cooling is consistent with a possible mastoid contribution to vestibular thermal buffering. Importantly, this study was not designed to test the thermal buffering hypothesis prospectively, and its observations are used here as a post hoc reinterpretation consistent with our model rather than confirmatory evidence. Reliance on this single study is a recognized limitation (see Section 4.7). The findings are nonetheless consistent with Prediction 1 of this hypothesis.

### 4.2. Physiological Evidence: Selective Brain Cooling Studies

Intranasal cooling studies show that the nasal/sinus route can influence intracranial temperature [13,14]. Animal research demonstrates that selective brain cooling of 2–4°C can be achieved via the nasal route, while core body temperature remains unchanged. These results confirm the anatomical heat-exchange pathway. Whether physiological variations in ambient temperature produce clinically meaningful intracranial thermal effects via this pathway remains to be tested and cannot be assumed from these experimental data.

### 4.3. Anatomical Evidence: Climate Correlation Reinterpreted

The inverse climate–pneumatization correlation (smaller sinuses in cold climates), previously interpreted as evidence against thermal function [2,3], is consistent with bidirectional adaptation: in cold climates, smaller air spaces reduce thermal windows that would dissipate core body heat; in warm climates, larger air spaces provide greater evaporative surface area for heat dissipation. This pattern may represent adaptation to the dominant thermal challenge in each environment. However, we acknowledge that this reinterpretation is correlational and that phylogenetic confounding, population-level differences in facial architecture (brachycephalic versus dolichocephalic morphology), and genetic drift may contribute to observed climate–pneumatization correlations independently of thermal selection pressure. We therefore treat this evidence as consistent with rather than directly supporting the hypothesis, and note that Rae et al. [15] have argued against simple cold-adaptation in Neanderthal sinus morphology, highlighting the complexity of inferring function from population-level anatomy.

### 4.4. Why Sensory Organs Rather than the Brain?

Brain parenchyma exhibits strong intrinsic thermoregulation, including high blood perfusion (50–60 mL/100 g/min), autoregulation, a CSF thermal buffer (150 mL), and multiple alternative cooling pathways [16]. In contrast, the vitreous and endolymph have zero perfusion, extremely low thermal inertia, and lack intrinsic regulation. A quantitative vulnerability analysis comparing thermal time constants for vitreous/endolymph versus brain parenchyma (Supplementary Materials S1, Section S9.4) indicates that these avascular structures are approximately 4- to 10-fold (conservatively 4- to 5-fold) more susceptible to thermal perturbation than brain tissue, making them likely primary targets for pneumatic thermal protection.

### 4.5. Ocular Implications

Although direct measurements of vitreous temperature during ambient cold exposure are currently unavailable in humans, the vitreous is known to exhibit temperature-dependent changes in viscosity and refractive index consistent with its gel composition [17]. These physical properties raise the theoretical possibility that brief thermal perturbations could influence light-scattering dynamics or vitreous microstructural motion. Anecdotal clinical observations, such as transient blurring or increased perception of floaters during cold exposure, may reflect subtle thermal changes in the vitreous environment, though this remains unverified. If the proposed thermoregulatory model is correct, inter-individual differences in periorbital pneumatization could contribute to variation in sensitivity to cold-induced visual disturbances. This represents a falsifiable clinical prediction that has not previously been considered in the literature.

### 4.6. Vestibular Implications

The vestibular endolymph is demonstrably the most thermally sensitive structure discussed in this hypothesis: caloric testing exploits a mere 7 °C temperature difference to reliably elicit nystagmus within 20–40 s [9], confirming that endolymph temperature responses are both rapid and clinically significant. The mastoid air cell system surrounds the vestibular labyrinth with only 2–8 mm separation through the otic capsule, providing a spatial arrangement analogous to the periorbital enclosure of the paranasal sinuses. Clinical observations from Švagan et al. (2025) [6] are consistent with a mastoid contribution to vestibular thermal buffering: after mastoidectomy, pressure-buffering capacity was preserved while vestibular thermal insulation was reduced under extreme thermal stimulation, with the authors characterizing the long-term clinical impact as minor. We therefore propose that mastoid pneumatization functions as a dedicated thermal shield for vestibular endolymph homeostasis, with Component 2 (mucosal heat delivery) and Component 3 (evaporative cooling) operating through mastoid mucosa in parallel with their sinonasal counterparts protecting the vitreous.

### 4.7. Limitations

This hypothesis has several important limitations. First, no study has directly measured vitreous or endolymph temperature during natural ambient thermal exposure in humans; all supporting evidence is indirect or inferential. Second, the sole clinical study consistent with the model (Švagan et al., 2025 [6]) was not designed to test this hypothesis prospectively, and independent replication is required. Third, the sinonasal contribution to respiratory warming (20–40%) is an approximation rather than a directly measured value, constituting the primary source of quantitative uncertainty in Component 2. Fourth, the lumped-parameter thermal model provides order-of-magnitude estimates; finite-element modeling with patient-specific anatomy would be required for precise spatial predictions. Fifth, climate-pneumatization correlations are subject to phylogenetic confounding, population-level differences in craniofacial architecture, and genetic drift, which may explain observed variation independently of thermal selection. Sixth, the model does not account for pathological states (sinusitis, mastoiditis) that alter air space volume, mucosal blood flow, and humidity gradients. Seventh, animal model data on intranasal brain cooling used extreme conditions not representative of physiological temperature variation, limiting direct relevance. Eighth, the proposed thermal function is not mutually exclusive with other established roles of pneumatized spaces (resonance, weight reduction, pressure regulation, nitric oxide production) and should be considered an additional functional dimension.

### 4.8. Testable Predictions

The following predictions are meant to be testable. Not confirming any prediction would weaken certain parts of the hypothesis while possibly keeping others intact.

#### 4.8.1. Prediction 1: Structural–Thermal Correlation

Pneumatization volume should inversely correlate with temperature variability around the eyes and ears during thermal stress. Proposed test: CT-based volumetry in 90 subjects divided by pneumatization tertile, with IR thermography during a standardized cold/heat challenge. Expected: r = −0.5 to −0.8.

#### 4.8.2. Prediction 2: Directional Asymmetry

Cold protection capacity should exceed heat dissipation by 5- to 15-fold. Proposed test: Measure respiratory heat delivery during cold exposure and evaporative water loss during heat exposure. Expected ratio: 5- to 15-fold (consistent despite parameter variability; see Supplementary Materials S1, Section S8).

#### 4.8.3. Prediction 3: Autonomic Modulation

Pharmacological intervention should selectively impact active components. Proposed test: Vasoconstrictors should decrease cold protection (Component 2) without affecting passive insulation (Component 1). Dehydration should reduce heat dissipation (Component 3). Expected outcome: Targeted components should be selectively impaired.

#### 4.8.4. Prediction 4: Clinical Phenotype

Loss of pneumatization should affect the thermal stability of sensory organs more than brain temperature. Proposed test: Assess post-FESS patients with periorbital thermography and caloric testing compared to EEG and cognitive measures. Expected outcome: Sensory effects should surpass neural effects.

#### 4.8.5. Prediction 5: Comparative Anatomy

Cross-species analysis should reveal a stronger link between pneumatization and eye/vestibular parameters than with brain mass. Proposed test: Phylogenetically controlled regression across 30–50 mammalian species. Expected outcome: Eye diameter and vestibular volume will more accurately predict pneumatization than brain mass.

## 5. Conclusions

We suggest that the paranasal sinuses and mastoid air cells form a three-part craniofacial thermal system that shields temperature-sensitive avascular structures, such as the vitreous and endolymph, from environmental thermal changes.

**The three components consist of:** (1) Passive insulation providing roughly 15-fold greater thermal resistance than bone; (2) active cold protection with an estimated capacity of 2–5 W; (3) active heat dissipation with an estimated capacity of 0.3–0.5 W. The 5- to 15-fold asymmetry favoring cold protection reflects thermodynamic constraints rather than design flaws.

**Novel contributions:** This framework (1) identifies avascular sensory organs as primary targets for protection based on vulnerability analysis; (2) distinguishes three mechanistically distinct components; (3) integrates the sinuses and mastoid into a unified system; (4) generates quantitative, testable predictions; and (5) reinterprets climate correlations as consistent with rather than contradictory to thermal function.

**Clinical implications (if validated):** Thermal-aware surgical planning may be necessary; postoperative cold sensitivity symptoms might have a physiological rather than psychological cause; protective strategies could be devised for at-risk patients.

We emphasize that this remains a hypothesis requiring thorough experimental testing. The significance lies not in claiming certainty but in providing a testable framework that can inspire new research directions. We encourage the scientific community to test these predictions, understanding that even partial validation would improve understanding, while refutation would clarify the true roles of these mysterious structures.

## Data Availability

The original contributions presented in this study are included in the Supplementary Materials. Further inquiries can be directed to the corresponding author.

## Supplementary Materials

The following supporting information can be downloaded at: https://www.mdpi.com/article/10.3390/jcm15114259/s1, Supplementary Materials S1: detailed calculations, parameter justifications, and sensitivity analysis, including thermal property tables, full derivations for the three thermal components, the asymmetric capacity ratio analysis, temporal dynamics, a comprehensive sensitivity analysis with Monte Carlo simulation, and internal validation cross-checks.

## Abbreviations

The following abbreviations are used in this manuscript:

CT	Computed tomography
IR	Infrared (thermography)
FESS	Functional endoscopic sinus surgery
CSF	Cerebrospinal fluid
