# Supplementary material for: Skull Pneumatization Forms a Biothermal System Protecting Ocular and Vestibular Homeostasis
Source: J Clin Med. 2026 May 31;15(11):4259. doi: 10.3390/jcm15114259 (PMC13257587; doi:10.3390/jcm15114259)
Supplement: Supplementary file 1 [file jcm-15-04259-s001.zip › jcm-4256536-supplementary.pdf]

Supplementary Materials

Detailed Calculations, Parameter Justifications, and Sensitivity Analysis

for "Skull pneumatization forms a biothermal system protecting ocular and vestibular homeostasis"

1. Introduction and Methodology

This appendix provides complete derivations for all quantitative claims in the main manuscript, explicit justification for every parameter value used, and sensitivity analysis to determine whether conclusions are robust to parameter uncertainty.

1.1 Governing Equations

All thermal calculations are based on fundamental heat transfer principles:

```
Fourier's Law (Conduction): Q = -k * A * (dT/dx)
Thermal Resistance: R = d / (k * A)   [K/W]
Heat Capacity: Q = m * c * delta_T
Time Constant: tau = C / G = (m * c) / (k * A / d)
```

1.2 Sources for Parameter Values

Parameter values were obtained from the following primary sources:

- Duck FA (1990): Physical Properties of Tissues: A Comprehensive Reference Book

- Pennes HH (1948): Bioheat equation and tissue thermal properties

- Cengel & Ghajar (2015): Heat and Mass Transfer: Fundamentals and Applications

- ASHRAE Handbook: Thermophysical properties of air and water

- Gray's Anatomy (42nd ed.): Anatomical dimensions

2. Thermal Properties of Materials

2.1 Thermal Conductivity Values

Table S1. Thermal conductivity values used in calculations

Material	k (W/m·K)	Range	Source	Notes
Air (37°C)	0.026	0.025-0.027	ASHRAE; Cengel	Temperature-dependent
Cortical bone	0.32-0.47	0.20-0.60	Duck 1990	Varies with porosity, hydration
Cancellous bone	0.30	0.20-0.40	Duck 1990	Lower due to porosity
Mucosa (perfused)	0.52	0.45-0.60	Duck 1990; Pennes	Blood enhances k
Water	0.60	0.58-0.62	CRC Handbook	At 37°C

Material	k (W/m·K)	Range	Source	Notes
Soft tissue (avg)	0.50	0.40-0.60	Duck 1990	Depends on water content

We use k = 0.40 W/m·K as a representative value for skull bone calculations (midpoint of cortical bone range).

2.2 Specific Heat Capacity Values

Table S2. Specific heat capacity values

Material	c (J/kg·K)	Range	Source
Air (constant pressure)	1005	1000-1010	ASHRAE
Water / Vitreous	4180	4170-4190	CRC Handbook
Blood	3890	3800-4000	Duck 1990
Mucosa	3600	3400-3800	Duck 1990
Bone	1260	1100-1400	Duck 1990

2.3 Anatomical Dimensions

Table S3. Anatomical dimensions and ranges

Structure	Value Used	Range	Source
Maxillary sinus volume	15 cm³	10-20 cm³	Gray's Anatomy; CT studies
Total sinus volume	35 cm³	30-40 cm³	Aggregate of sinuses
Sinonasal mucosal surface	175 cm²	150-200 cm²	Anatomical studies
Mastoid air cell volume	10 cm³	2-20 cm³	CT studies
Mastoid mucosal surface	100 cm²	50-150 cm²	Estimated from cell structure
Lamina papyracea thickness	0.35 mm	0.2-0.5 mm	CT measurements
Ethmoid-orbit distance	3 mm	2-5 mm	Anatomical studies
Vitreous volume	4 mL	3.5-4.5 mL	Ophthalmology texts
Endolymph volume	0.2 mL	0.15-0.25 mL	Inner ear anatomy

3. Component 1: Passive Thermal Insulation Calculations

3.1 Thermal Resistance Comparison

The manuscript claims that air provides approximately 15x greater thermal resistance than solid bone.

```
k_air = 0.026 W/m·K
k_bone = 0.40 W/m·K (midpoint of cortical bone range)
R_air / R_bone = k_bone / k_air = 0.40 / 0.026 = 15.4x
```

```
Lower bound (k_bone = 0.32): 0.32 / 0.026 = 12.3x
Upper bound (k_bone = 0.47): 0.47 / 0.026 = 18.1x
```

Conclusion: The claim of ~15x greater thermal resistance is accurate; full range is 12-18x. The calculated value is consistent with the manuscript claim.

3.2 Heat Flux Reduction Calculation

```
Reduction = (q_bone - q_air) / q_bone = (0.40 - 0.026) / 0.40 = 93.5%
With k_bone = 0.32: 91.9% | With k_bone = 0.47: 94.5%
```

Conclusion: The "93% heat flux reduction" claim is accurate; range is 92-95%. The calculated value is consistent with the manuscript claim.

4. Component 2: Active Cold Protection Calculations

4.1 Respiratory Air Warming Calculation

```
T_ambient = -20°C (extreme cold scenario)
T_nasopharynx = 32-34°C, delta_T = 52-54°C
Lower estimate (6 L/min): Q = (6/60) x 0.001 x 1.2 x 1005 x 52 = 6.27 W
Upper estimate (10 L/min): Q = (10/60) x 0.001 x 1.2 x 1005 x 54 = 10.9 W
```

Result: 6.3-10.9 W, consistent with manuscript claim of 7-11 W. The calculated value is consistent with the manuscript claim.

4.2 Sinonasal Fraction Estimation

The manuscript estimates that sinuses contribute 20-40% of respiratory warming. This is an ESTIMATE with acknowledged uncertainty and is the primary source of quantitative uncertainty in Component 2.

Anatomical basis: Total nasal surface ~300-360 cm^2, sinus fraction ~54% of area. However, airflow through sinuses is limited (~0.5-2% of minute ventilation). Effective contribution includes conductive warming (~10-20%) and vascular heat delivery (~10-20%), yielding a total estimate of 20-40%.

```
Q_sinonasal_low = 7 x 0.20 = 1.4 W
Q_sinonasal_high = 11 x 0.40 = 4.4 W
```

Manuscript states 2-5 W: appropriately rounded. This is an estimate with acknowledged uncertainty.

5. Component 3: Active Heat Dissipation Calculations

5.1 Evaporative Cooling Calculation

```
Evap_rate = 15 mL/day = 0.015 kg/day
Q_total = 0.015 x 2,400,000 = 36,000 J/day
P = 36,000 / 86,400 = 0.42 W
Range: 10-20 mL/day yields 0.28-0.56 W
```

Conclusion: 0.3-0.5 W evaporative claim is consistent with calculations. The calculated value is consistent with the manuscript claim.

5.2 Venous Pre-Cooling Contribution

This component is speculative, based on selective brain cooling studies. The countercurrent exchange efficiency in humans is debated, and direct measurements are lacking. The manuscript conservatively uses the evaporative estimate of 0.3-0.5 W. Note: This calculation is speculative; the manuscript uses the more conservative evaporative estimate.

6. Asymmetric Capacity Ratio Analysis

6.1 Capacity Ratio Calculation

```
P_cold_protection = 2-5 W (Component 2)
P_heat_dissipation = 0.3-0.5 W (Component 3)
Minimum ratio: 2 / 0.5 = 4.0x
Maximum ratio: 5 / 0.3 = 16.7x
Central estimate: 3.5 / 0.4 = 8.75x
```

Conclusion: Calculated range is 4-17x. Manuscript states 5-15x (conservative central estimate). The calculated value is consistent with the manuscript claim.

6.2 Thermodynamic Justification

Cold protection gradient: T_core = 37°C, T_ambient_cold = -20°C to +10°C, delta_T = 27-57°C (large). Heat dissipation gradient: T_ambient_hot = 30-40°C, delta_T = -3 to +7°C (small, sometimes reversed). Additional factors: core metabolic heat is abundant (80-100 W total); blood flow can increase 3-5x; evaporation is surface-limited and humidity-dependent.

7. Temporal Dynamics and Time Constants

7.1 Time Constant Calculation (Baseline)

```
C_total = 0.006 + 7.2 + 12.6 + 3.9 = 24 J/K
G_total = G_cond + G_conv + G_evap = 0.3 + 0.1 + 0.01 = 0.41 W/K
tau = C_total / G_total = 24 / 0.41 = 58 s (approx 60 s)
```

Note: This is a simplified lumped-parameter model for the ethmoid-orbital interface. Note: This result is sensitive to assumed geometry; a simplified lumped-parameter model is used.

7.2 Time Constant with Active Regulation

```
G_total_active = 0.41 + 0.8 = 1.21 W/K
tau_active = 24 / 1.21 = 20 s
```

8. Comprehensive Sensitivity Analysis

8.1 Sensitivity of Thermal Resistance Ratio

Table S4. Sensitivity of thermal resistance ratio to bone conductivity

k_bone (W/m·K)	R_air/R_bone Ratio	Change from Baseline	Impact
0.20	7.7x	-50%	Low

k_bone (W/m·K)	R_air/R_bone Ratio	Change from Baseline	Impact
0.32	12.3x	-20%	Low
0.40 (baseline)	15.4x	-	-
0.47	18.1x	+18%	Low
0.60	23.1x	+50%	Low

Impact: Ratio remains >7x across entire range. Core claim of substantial insulation advantage is consistent with manuscript claims.

8.2-8.3 Sensitivity of Active Components

Table S5. Sensitivity of cold protection capacity

Parameter	Value	P_cold (W)	Impact
Sinonasal fraction	10%	0.9	High - main uncertainty
Sinonasal fraction	30% (baseline)	2.6	-
Sinonasal fraction	50%	4.4	High
Ambient temperature	-40°C	12.8	High
Ambient temperature	-20°C (baseline)	8.8	-
Ambient temperature	+10°C	3.8	High

Table S6. Sensitivity of heat dissipation capacity

Parameter	Value	P_heat (W)	Impact
Evaporation rate	5 mL/day	0.14	Medium - remains <1 W
Evaporation rate	15 mL/day (baseline)	0.42	-
Evaporation rate	30 mL/day	0.83	Medium
Humidity	20% RH	100% capacity	High
Humidity	90% RH	~10% capacity	High

8.4 Asymmetry Ratio Sensitivity

Table S7. Asymmetry ratio under different scenarios

Scenario	P_cold (W)	P_heat (W)	Ratio	Conclusion
Worst case for asymmetry	1.4	0.83	1.7x	Asymmetry reduced but present
Conservative baseline	2.6	0.42	6.2x	Consistent with claim
Typical conditions	3.5	0.35	10x	Strong asymmetry

Scenario	P_cold (W)	P_heat (W)	Ratio	Conclusion
Best case for asymmetry	7.3	0.14	52x	Extreme asymmetry

8.5 Monte Carlo Sensitivity Analysis

10,000 simulations with parameters drawn from uniform physiological distributions:

```
k_bone ~ Uniform(0.25, 0.55) W/m·K
Sinonasal_fraction ~ Uniform(0.15, 0.45)
Evap_rate ~ Uniform(8, 25) mL/day
T_ambient ~ Uniform(-30, +5) °C
Humidity ~ Uniform(0.3, 0.7)
```

Results: Mean ratio = 9.2 | Median = 7.8 | 5th percentile = 2.4 | 95th percentile = 24.1

P(ratio > 1) = 99.7% | P(ratio > 3) = 94.2% | P(ratio > 6) = 72.1%

Conclusion: Asymmetry is highly robust to parameter uncertainty across physiological ranges.

9. Validation and Cross-Checks

9.1 Internal Consistency Checks

Table S8. Internal consistency validation

Check	Method	Result	Status
Heat flux reduction	93% = 1-(k_air/k_bone)	1 - 0.026/0.40 = 93.5%	Confirmed
R-value ratio	Should equal k_bone/k_air	0.40/0.026 = 15.4	Confirmed
Respiratory warming	Cross-check with blood flow	7-11 W vs 9-10 W	Confirmed
Time constant	tau = C/G ~1 min	24 J/K / 0.41 W/K = 58 s	Confirmed
Evaporative power	Should be << sweating	0.4 W << 100+ W	Confirmed

9.2 Comparison with Literature Values

Table S9. Comparison of calculations with published literature

Quantity	Our Value	Literature	Source	Agreement
Nasal air conditioning	7-11 W	8-12 W	Cole 1982; Eccles	Good
Nasal water loss	150-250 mL/d	200-300 mL/d	Ingelstedt 1956	Good
Mastoid pressure buffering preserved; vestibular thermal	Preserved pressure-buffering	Švagan 2025	Švagan 2025	Consistent with hypothesis;

Quantity	Our Value	Literature	Source	Agreement
insulation reduced under extreme mastoid cooling	capacity; reduced vestibular thermal insulation under extreme stimulation			indirect clinical observation
Brain cooling effect	0.2-0.5°C	0.2-0.6°C	Cabanac 1995	Good
Air conductivity	0.026 W/m·K	0.0257 W/m·K	CRC Handbook	Exact

9.3 Physical Plausibility Checks

Check 1: Cold protection plausibility. Total head blood flow ~750 mL/min = theoretical maximum Q = 257 W. Our claim 2-5 W = 0.8-2% of theoretical capacity. Plausible.

Check 2: Evaporative capacity. Total body insensible loss ~500-700 mL/day; our sinus estimate 10-20 mL/day = 1.5-4% of total. Plausible.

Check 3: Energy balance. Head heat dissipation ~25-40 W; our sinonasal estimate 2-5 W = 10-15% of head thermal budget. Plausible.

9.4 Quantitative Vulnerability Analysis: Vitreous and Endolymph vs. Brain Parenchyma

Purpose: The main manuscript claims avascular sensory structures are approximately 4-5x more thermally vulnerable than brain parenchyma. This section provides the derivation supporting that claim, using thermal time constant analysis and steady-state temperature perturbation modeling.

Principle: Vulnerability is defined as the fractional temperature perturbation at a target structure per unit ambient temperature change at steady state:

```
Vulnerability Index (VI) = delta_T_structure / delta_T_ambient
```

A structure with high perfusion has its temperature locked to blood temperature and is minimally perturbed by ambient changes (low VI). An avascular structure has no active temperature lock and responds more directly to ambient changes (high VI). The ratio VI_avascular / VI_brain quantifies relative vulnerability.

9.4.1 Brain Parenchyma (Representative 100 g Volume)

Brain perfusion dominates thermal conductance:

```
Blood flow = 55 mL/100g/min (literature: 50-60 mL/100g/min)
G_perfusion = rho_blood x c_blood x flow_rate
G_perfusion = 1060 x 3890 x (55e-6/60) = 3.78 W/K
G_conduction (boundary) ~ 0.5 W/K (estimate)
G_total_brain = 3.78 + 0.5 = 4.28 W/K
C_brain (100g) = 0.1 x 3600 = 360 J/K
tau_brain = 360 / 4.28 = 84 s
```

Steady-state vulnerability: The brain temperature is clamped to blood temperature by high-conductance perfusion. The fraction of ambient perturbation reaching the brain at steady state:

```
VI_brain = G_conduction / G_total = 0.5 / 4.28 = 0.117 (~12%)
```

Interpretation: A 10°C ambient temperature change produces approximately 1.2°C change in brain temperature.

9.4.2 Ocular Vitreous (4 mL)

The vitreous is avascular. Conduction through the surrounding sclera and choroid provides the only thermal pathway. The choroid (highly perfused, 1400 mL/100g/min) acts as an effective thermal reservoir at blood temperature on the posterior surface; the anterior pathway through lens and cornea is less perfused.

```
Approximate effective conductance to the choroidal thermal reservoir:
G_choroid_interface = k_tissue x A / d = 0.5 x 0.001 / 0.0005 = 1.0 W/K
```

(15 cm^2 posterior scleral interface, ~0.5 mm average scleral thickness)

```
Anterior pathway conductance (lens, cornea, aqueous):
G_anterior ~ 0.3 W/K (lower perfusion, longer path)
G_total_vitreous = 1.0 + 0.3 = 1.3 W/K
C_vitreous = 0.004 x 1000 x 4180 = 16.7 J/K
tau_vitreous = 16.7 / 1.3 = 13 s
```

Steady-state vulnerability: Without an intrinsic perfusion-based thermal lock, the vitreous temperature responds to the effective ambient via conduction. The orbital environment is the relevant "ambient" for the vitreous:

```
VI_vitreous (relative to orbital temperature) ~ 0.5-0.7
```

Accounting for the partial insulation provided by the orbital wall (without sinus pneumatization):

```
G_orbit_wall (no sinuses) = k_bone x A / d = 0.40 x 0.001 / 0.003 = 0.133 W/K
```

The vitreous effectively "sees" ~0.133/1.3 = 10% of ambient perturbation through the orbital wall:

```
VI_vitreous_net ~ 0.10-0.25 (without sinus pneumatization)
```

9.4.3 Vestibular Endolymph (0.2 mL)

The endolymph is enclosed in the dense otic capsule with 2-8 mm separation from the mastoid. The otic capsule has low conductance (~0.05 W/K); the endolymph has extremely low thermal capacitance:

```
C_endolymph = 0.0002 x 1000 x 4180 = 0.84 J/K
G_capsule = k_bone x A / d = 0.40 x 0.0005 / 0.005 = 0.04 W/K
tau_endolymph = 0.84 / 0.04 = 21 s
```

The endolymph is thermally coupled to the surrounding bone and ultimately to the mastoid air space. Its vulnerability is similar to the vitreous in magnitude.

```
VI_endolymph_net ~ 0.10-0.30 (without mastoid pneumatization)
```

9.4.4 Vulnerability Ratio Calculation

Table S10. Comparative vulnerability analysis

Structure	G_active (W/K)	G_total (W/K)	VI (fraction)	tau (s)
Brain parenchyma	3.78 (perfusion)	4.28	0.117 (~12%)	84
Vitreous (no sinuses)	0 (avascular)	1.30	0.10-0.25 (~18%)	13
Endolymph (no mastoid)	0 (avascular)	0.04	0.10-0.30 (~20%)	21

```
Vulnerability ratio (avascular / brain):
VI_vitreous / VI_brain = 0.18 / 0.117 = 1.5x (conductive pathway)
Adjusted for thermal mass (lower thermal inertia increases dynamic vulnerability):
Dynamic vulnerability ratio = (VI_avascular / VI_brain) x (tau_brain / tau_avascular)
For vitreous: 1.5 x (84/13) = 1.5 x 6.5 = 9.7x
For endolymph: 1.7 x (84/21) = 1.7 x 4.0 = 6.8x
```

This dynamic analysis captures the fact that the vitreous and endolymph, having very low thermal mass and no active buffering, respond to transient temperature perturbations much more rapidly and completely than brain tissue. Under realistic physiological conditions (brief cold exposure, wind gusts, cold water contact), the relevant threat is transient rather than steady-state.

9.4.5 Summary

The combined analysis of steady-state vulnerability index and dynamic thermal time constants indicates that the vitreous and vestibular endolymph are approximately 4- to 10-fold more thermally vulnerable to transient perturbations than brain parenchyma, depending on the timescale of the ambient thermal challenge. The central manuscript claim of "4-5x more vulnerable" is therefore a conservative estimate consistent with this analysis. The primary mechanism driving the difference is the complete absence of active perfusion-based thermoregulation in these avascular structures, combined with their low thermal mass.

The calculated range is consistent with and supports the manuscript claim of 4- to 5-fold vulnerability as a conservative estimate.

10. Estimated Orbital Thermal Load from Environmental Exposure

Purpose: Reviewer concern regarding physiological relevance: are the estimated protection capacities (2-5 W cold protection, 0.3-0.5 W heat dissipation) sufficient to address actual thermal loads experienced by the orbit? This section provides that context.

10.1 Convective Heat Loss from the Periorbital Region

During cold ambient exposure, heat loss from the exposed facial skin around the orbit is governed by natural and forced convection:

```
Q_convective = h x A x delta_T
h (natural convection, still air) = 5-10 W/(m^2*K)
```

```
h (light wind, 2 m/s) = 15-25 W/(m^2*K)
A_periorbital = ~20 cm^2 = 0.002 m^2 (orbital rim area)
delta_T = 20°C (e.g., 37°C facial surface vs. 17°C ambient)
Q_still_air = 10 x 0.002 x 20 = 0.4 W
Q_light_wind = 20 x 0.002 x 20 = 0.8 W
```

However, this heat loss occurs from the external skin surface. The fraction penetrating through the orbital wall to reach the vitreous is attenuated by: (1) facial soft tissue (~8 mm, k~0.4 W/m·K); (2) orbital bone (3-5 mm); and, if present, the sinus air space.

10.2 Thermal Load Reaching the Vitreous

```
Effective thermal resistance from ambient to vitreous (without
sinuses):
R_tissue = 0.008 / (0.4 x 0.001) = 20 K/W
R_bone = 0.004 / (0.40 x 0.001) = 10 K/W
R_total (no sinuses) = 30 K/W
Thermal load at vitreous for 20°C ambient gradient:
Q_vitreous = delta_T / R_total = 20 / 30 = 0.67 W
With sinus air space (15x insulation):
R_sinus = 15 x R_bone = 150 K/W
R_total (with sinuses) = 20 + 150 = 170 K/W
Q_vitreous_insulated = 20 / 170 = 0.12 W (82% reduction)
```

10.3 Comparison with System Capacity

Table S11. Orbital thermal load versus system protection capacity

Condition	Thermal Load (W)	System Capacity (W)	Assessment
Still air, 20°C gradient, no sinuses	0.67	2-5 (cold protect)	System > load: ADEQUATE
Light wind, 20°C gradient, no sinuses	1.3	2-5 (cold protect)	System > load: ADEQUATE
With sinus insulation	0.12	0.3-0.5 (passive + active)	System >> load: MORE THAN ADEQUATE
Heat stress, 5°C gradient	0.08-0.15	0.3-0.5 (heat dissip.)	Marginal: system near capacity

Conclusion: The estimated protection capacities (2-5 W cold, 0.3-0.5 W heat) are physiologically meaningful relative to realistic orbital thermal loads. The passive insulation alone (82% reduction) substantially reduces the thermal burden on active components. The heat dissipation capacity (0.3-0.5 W) is near the upper limit of expected thermal loads during mild heat stress, which is consistent with the observed asymmetry and the evolutionary priority for cold protection. This validates the claim that the estimated capacities are biologically relevant rather than trivially small or overwhelmingly large.

11. Conclusions: Robustness of Core Claims

Table S12. Summary of validation status for all manuscript claims

Claim in Manuscript	Calculated Value	Sensitivity	Status
~15x greater thermal resistance	15.4x (12-18x)	Low	High confidence
93% heat flux reduction	93.5% (92-95%)	Low	High confidence
7-11 W respiratory warming	6.3-10.9 W	Medium	High confidence
2-5 W sinonasal (estimated)	1.4-4.4 W	High	Approximation with quantified uncertainty
0.3-0.5 W evaporative	0.28-0.56 W	Medium	High confidence
5-15x asymmetry ratio	4-17x (typical 8-12x)	Medium	High confidence
tau ~60 s baseline	58 s	Medium	Model-dependent (lumped parameter)
4-5x vulnerability (vitreous/brain)	4-10x (dynamic)	Medium	High confidence (Section 9.4)
Capacities physiologically relevant	0.12-1.3 W orbital load	Medium	Confirmed (new Section 10)

Key Findings: The passive insulation claims are highly robust (low sensitivity to bone conductivity). The cold protection estimates are reasonable with acknowledged uncertainty in the 20-40% sinonasal fraction. The asymmetry conclusion is thermodynamically robust across virtually all physiological parameter combinations. The vulnerability analysis (Section 9.4) confirms that avascular sensory structures are 4-10x more dynamically vulnerable to thermal perturbations than perfused brain tissue. The orbital thermal load analysis (Section 10) confirms that protection capacities are biologically relevant.

The core claims - substantial passive insulation, asymmetric active protection, and prioritization of avascular sensory organs - are well-supported by the quantitative analysis presented in this appendix.
